# Complement Mediated Hemolytic Anemias in the COVID-19 Era: Case Series and Review of the Literature

**DOI:** 10.3389/fimmu.2021.791429

**Published:** 2021-11-25

**Authors:** Bruno Fattizzo, Raffaella Pasquale, Valentina Bellani, Wilma Barcellini, Austin G. Kulasekararaj

**Affiliations:** ^1^ Hematology Unit, Fondazione IRCCS Ca’ Granda Ospedale Maggiore Policlinico, Milan, Italy; ^2^ Department of Oncology and Hemato-Oncology, University of Milan, Milan, Italy; ^3^ Hematology Unit, King’s College Hospital, London, United Kingdom

**Keywords:** paroxysmal nocturnal hemoglobinuria, cold agglutinin disease, SARS-CoV-2, COVID19 vaccine, hemolytic uremic syndrome, autoimmune hemolytic anemia

## Abstract

The complex pathophysiologic interplay between SARS-CoV-2 infection and complement activation is the subject of active investigation. It is clinically mirrored by the occurrence of exacerbations of complement mediated diseases during COVID-19 infection. These include complement-mediated hemolytic anemias such as paroxysmal nocturnal hemoglobinuria (PNH), autoimmune hemolytic anemia (AIHA), particularly cold agglutinin disease (CAD), and hemolytic uremic syndrome (HUS). All these conditions may benefit from complement inhibitors that are also under study for COVID-19 disease. Hemolytic exacerbations in these conditions may occur upon several triggers including infections and vaccines and may require transfusions, treatment with complement inhibitors and/or immunosuppressors (i.e., steroids and rituximab for AIHA), and result in thrombotic complications. In this manuscript we describe four patients (2 with PNH and 2 with CAD) who experienced hemolytic flares after either COVID-19 infection or SARS-Cov2 vaccine and provide a review of the most recent literature. We report that most episodes occurred within the first 10 days after COVID-19 infection/vaccination and suggest laboratory monitoring (Hb and LDH levels) in that period. Moreover, in our experience and in the literature, hemolytic exacerbations occurring during COVID-19 infection were more severe, required greater therapeutic intervention, and carried more complications including fatalities, as compared to those developing after SARS-CoV-2 vaccine, suggesting the importance of vaccinating this patient population. Patient education remains pivotal to promptly recognize signs/symptoms of hemolytic flares and to refer to medical attention. Treatment choice should be based on the severity of the hemolytic exacerbation as well as of that of COVID-19 infection. Therapies include transfusions, complement inhibitor initiation/additional dose in the case of PNH, steroids/rituximab in patients with CAD and warm type AIHA, plasma exchange, hemodialysis and complement inhibitor in the case of atypical HUS. Finally, anti-thrombotic prophylaxis should be always considered in these settings, provided safe platelet counts.

## Introduction

There is an increasing interest in the relationship between COVID-19 infection and complement activation. Several reports highlighted that the virus is able to induce an over inflammatory state encompassing the activation of various pathways such as coagulation (namely thrombo-inflammation) and the complement cascade (1–3). The latter is a complex system which may be activated by three different ways: the classical, the lectin and the alternative pathway. The former two require the presence of a specific trigger, either an immune-complex or an infectious agent. The complement alternative pathway is homeostatically active and its functioning may be amplified in the presence of triggers. Complement-mediated hematologic diseases include paroxysmal nocturnal hemoglobinuria (PNH), autoimmune hemolytic anemia (AIHA), particularly cold agglutinin disease (CAD), and hemolytic uremic syndrome (HUS). PNH is caused by the acquisition of somatic mutation of *PIG-A* gene resulting in the loss of glycosylphosphatidylinositol-anchored proteins, including the complement inhibitors CD55 and CD59. PNH cells are therefore subject to complement-mediated destruction that mainly involves erythrocytes with consequent hemolytic anemia. PNH is managed successfully with terminal complement inhibitors targeting C5 (eculizumab or ravulizumab) with good control of intravascular hemolysis (IVH), but potentially inducing iatrogenic extravascular hemolysis (EVH) which is targeted by novel C3 inhibitors (4). CAD is an autoimmune hemolytic anemia caused by cold reactive anti-erythrocyte autoantibodies usually of the IgM class that are intrinsically able to fix complement with consequent positivity of the direct antiglobulin test (DAT) for C3d (5). This may result in both IVH and EVH due to either terminal activation of complement and membrane attack complex (MAC) formation, or C3b deposition on erythrocytes and reticulo-endothelial phagocytosis. CAD is mainly treated with immunosuppressants (steroids and the anti-CD20 monoclonal antibody rituximab) and complement inhibitors are in clinical trials with promising results (6). Complement activation may be observed even in warm type AIHA (wAIHA), particularly in case of high levels of IgG anti-erythrocyte autoantibodies, resulting in DAT positivity for IgG+C. IgG+C wAIHAs are generally more severe and show higher relapse frequency. Clinical trials with complement inhibitors in wAIHA are also ongoing (7). HUS is a thrombotic microangiopathy characterized by the formation of platelet microthrombi in arterioles and capillaries, causing platelet consumption, nonimmune hemolytic anemia, and acute renal injury. Typical HUS is caused by Shiga-like toxin (verotoxin) produced by Escherichia coli (O157: H7) and Shiga toxin by Shigella dysenteriae. The atypical form may be secondary to bacteria, medication, or immune processes capable of endothelial damage, or to congenital or acquired conditions inducing widespread complement activation, such as atypical familial HUS. In the latter cases C5 inhibitors are employed with clinical benefit (8). All these complement-mediated hemolytic conditions may experience exacerbations upon several triggers including infections, surgery, traumas (4, 5). Particularly, hemolytic flares in PNH patients on complement inhibitors are denominated breakthrough hemolysis (BTH) (4). COVID19 infection and its vaccine are also possible triggers through various mechanisms including lectin- and alternative pathway activation by Nucleocapside and Spike viral proteins, molecular mimicry and autoantibody production with activation of the classical pathway, etc (1). In this manuscript we describe four patients who experienced PNH BTH and CAD exacerbation after either COVID infection or SARS-Cov2 vaccine and we review the most recent literature.

## Methods

We report four patients, 2 with PNH and 2 with CAD, diagnosed according to the current guidelines (4, 5) and regularly followed at a tertiary university hospital in Milan, Italy, who experienced hemolytic flares during COVID-19 infection or after SARS-CoV-2 vaccine.

The study was conducted according the Declaration of Helsinki and approved by the local Ethical Committee. Patients signed informed consent.

We conducted a review of the literature about complement mediated hemolytic anemias and COVID-19 infection and SARS-CoV-2 vaccine by searching for indexed articles and published abstracts until September 2021 in MEDLINE *via* PubMed and the National Library of Medicine. The following keywords were used: paroxysmal nocturnal hemoglobinuria; cold agglutinin disease; SARS-CoV-2; COVID19 vaccine; hemolytic uremic syndrome; autoimmune hemolytic anemia; and complement. We selected only studies including human subjects with a clear diagnosis of PNH, CAD, AIHA, HUS, and reporting data on treatment and outcome of COVID-19 infection and of the hemolytic flare.

## Results

### PNH Flares During COVID-19 Infection

#### Case Description

A 27-year-old man was diagnosed with PNH in 2010 (granulocyte clone size 93%) associated with transfusion dependent anemia (Hb 6.8 g/dL, LDH 7.5 x upper limit of normality, ULN, PLT 121x10^9^/L, neutrophils 1.8 x10^9^/L, reticulocytes 121x10^9^/L) and symptoms of hemolysis with abdominal pain. He was immediately started on eculizumab 900 mg fortnightly with good response (Hb 11.1 g/dL, LDH 1.1xULN, reticulocytes 51x10^9^/L). In March 2021, a nasopharyngeal swab for SARS-Cov-2 performed for screening purposes was positive and the patient was initially asymptomatic. Two days later, he presented to the emergency room complaining of shortness of breath, asthenia, and dark urine. Hb was 6.1 g/dL and LDH increased to 10.7 x ULN. A CT scan revealed bilateral interstitial pneumonia and he was admitted to hospital and treated with antibiotics, dexamethasone, and low molecular weight heparin (LMWH), as well as an earlier dose of eculizumab (10 days after the previous one). During admission he also required red blood cells (RBC) transfusion (3 units) and progressively recovered.

#### Literature Review

**Table 1** summarizes a literature review of PNH cases experiencing hemolytic flares after COVID-19 infection. Since March 2020, we found eight case reports describing a total of 19 patients, 15 with classic hemolytic PNH and 4 with PNH in the context of an aplastic anemia (9–16). Patients were mostly women (63% females, 37% males), with a wide age range (19 to 77 years). 15 patients were on active therapy with a complement inhibitor, and four were treatment naïve. The great majority (89.4%) experienced a mild to moderate COVID-19 infection, whilst only two subjects needed intensive care unit admission and one died of respiratory failure (11, 14). PNH flares had heterogenous severity with some patients requiring RBC transfusions and additional doses of complement inhibitors, and other experiencing only mild Hb reduction with slight LDH elevation. Importantly, no thrombotic episodes were reported. Specifically, Pike et al. reported the experience of the Leeds Paroxysmal Nocturnal Hemoglobinuria national service. Within their PNH cohort, they observed COVID-19 infection in 4 patients on anti-complement therapy (3 eculizumab, 1 ravulizumab); 75% experienced a BTH and one patient died from respiratory failure after being intubated, despite additional doses of eculizumab for ongoing haemolysis (14). Similarly, Barcellini et al. analyzed the experience of eight Italian PNH reference centers and reported 4 patients who had positive SARS-Cov-2 on routine surveillance SARS-Cov-2 nasal-pharyngeal swab. Only two subjects developed mild respiratory symptoms and only one experienced BTH. None of them required hospitalization (16). Additionally, Kulasekararaj et al. reported the clinical course, degree of intravascular hemolysis and outcomes of COVID-19 in four PNH patients, and showed a beneficial effect of complement inhibition both in controlling the intravascular haemolysis and in dampening the hyperinflammatory lung damage due to COVID-19 (13).

**Table 1:** Hemolytic flares in patients with paroxysmal nocturnal hemoglobinuria during COVID-19 infection.

PNH type	Therapy	N° of patients	COVID outcome	PNH outcome	Reference
Classic PNH	Eculizumab	1	Resolved	Clinical remission	*Schuller et al., Annals of Hematology 2021* (9)
Classic PNH	LMWH, dexamethasone, cefuroxime for COVID At the time of diagnosis of COVID19 the patient was waiting for approval of eculizumab	1	Resolved (test negative after 3 months)	Clinical remissionPNH clone did not change.	*Sokol et al., Case Rep Med 2021* (10)
Classic PNH	Antibiotics, Hydroxychloroquine Lopinavir/ritonavir Tocilizumab for COVID Eculizumab	1	Resolved	Clinical remission	*Genthon et al., Leukemia & Lymphoma 2021* (11)
AA PNH	Eculizumab (2 doses) then ravulizumab (2 doses)	1	Resolved	Persistence of pancytopenia	*Otieno et al., Case Rep Hematol 2021* (12)
Classic PNH	1) Ravulizumab 2) Eculizumab, blood transfusions 3) naïve to complement inhibitor treatment, warfarin 4) naïve to complement inhibitor treatment, warfarin	4	1)Resolved 2)Resolved 3)Resolved 4)Resolved	1)Resolved 2)Resolved 3)Resolved 4)Readmitted with worsening symptoms	*Kulasekararaj et al., Br J Haematol 2020* (13)
1) Classic PNH2) AA PNH3) AA PNH4) AA PNH	1) Ravulizumab 2) Eculizumab, antibiotics 3) Eculizumab, 4 units packed red cells, antibiotics 4) Eculizumab, 4 units packed red cells, antibiotics	4	1)Resolved 2)Resolved 3)Death 4)Resolved	1)Resolved2)Resolved3) Death4)Resolved	*Pike A et al., Br J Haematol 2020* (14)
Classic PNH	1) Eculizumab 2) Eculizumab 3) Eculizumab	3	1)Resolved 2)Resolved 3)Resolved	1)Resolved2)Resolved3)Resolved	*Araten et al., Am J Case Reports 2020* (15)
Classic PNH	1)Treatment naïve 2)Ravulizumab 3)Ravulizumab 4)Eculizumab	4	1)Resolved 2)Resolved 3)Resolved 4)Resolved	1)No BTH 2)No BTH 3)Resolved4)No BTH	*Barcellini W et al., Br J Haematol 2021* (16)

PNH, paroxysmal nocturnal hemoglobinuria; AA, aplastic anemia; BTH, breakthrough hemolysis; RBC, red blood cells; rhEPO, recombinant human erythropoietin.

Our case report along with the review of the literature suggest that PNH hemolytic flares may occur during COVID-19 infection and may be handled with transfusion support and additional doses of complement inhibitors. The latter, rather than increasing the risk of a more severe COVID-19 infection, appear to dampen the over-inflammatory state induced by SARS-CoV-2 (16, 17). Additionally, the role of complement activation in both early (18) and late stage (19) COVID-19 infection and the potential to target them with ravulizumab is currently being studied in two randomized controlled trials. The latter trial was interrupted early due to futility on interim analysis. The fact that no thrombotic events were observed during these acute hemolytic episodes suggest a further protective role of complement inhibition and may also be in keeping with the high level of medical surveillance/patient education in PNH patients.

### PNH Flares After SARS-CoV-2 Vaccination

#### Case Description

A 17-year-old man was diagnosed with classic hemolytic PNH in a low-income Country in 2019 and was managed with vitamin and iron support, warfarin prophylaxis, and RBC transfusions during hemolytic crises. In March 2021, he came to our center and was started on eculizumab with optimal control of intravascular hemolysis and warfarin was stopped. In July 2021, 5 days after the first dose of Pfizer mRNA vaccine (14 days after prior eculizumab dose), he experienced a decrease of 1.5 g/dL Hb (from 12.6 to 11.1 g/dL) associated with an increase in unconjugated bilirubin (1.9 to 3.2 mg/dL) and LDH levels (from normal to 2 x ULN). He complained of mild abdominal pain and urine darkening in the last 2 days. He received eculizumab treatment and symptoms progressively recovered soon after therapy. One-week later laboratory parameters were normalized.

#### Literature Review

mRNA-based vaccines act by inducing the expression of SARS-Cov-2 Spike protein in order to elicit immune recognition and antibody response. Yu et al. recently illustrated that Spike protein is able to activate complement alternative pathway by competing with complement factor H for binding heparin sulfate, thus representing a possible trigger for PNH reactivation (20). However, Gerber et al. recently showed that the addition of Spike protein didn’t increase hemolysis of PNH erythrocytes *ex vivo*, suggesting that hemolysis isn’t due to the direct effect of Spike protein on red blood cells but rather to the activation of the classic pathway after antibody generation (21). Clinically, 8 PNH patients experiencing hemolytic flares after SARS-Cov-2 vaccine have been reported, including 6 BTH and 2 exacerbations in treatment naïve subjects (**Table 2**) (21–23). Gerber et al. reported six patients with PNH, who received either Moderna or Pfizer-BioNTech vaccine. Four, all on intravenous ravulizumab and one in combination therapy with D inhibitor danicopan, showed signs of BTH from the vaccine day up to 5 days later, 2 with severe anemia, and 1 requiring transfusion support. Interestingly, only 1 patient who was therapy naïve experienced a likely thrombotic episode. Five days after the second dose of the Pfizer-BioNTech vaccine, he developed abdominal pain and CT scan showed peripancreatic fat stranding indicating possible small bowel microvascular thrombosis; D-dimer was elevated, and the patient was initiated on ravulizumab with signs and symptoms recovery (21). We previously described another case of PNH on treatment with subcutaneous ravulizumab displaying severe BTH the day after the second dose of Moderna vaccine. She was handled with supportive treatment and recombinant erythropoietin with progressive recovery. The latter, along with that reported in the present report, were the only 2 BTH episodes observed after SARS-CoV-2 vaccine in our cohort of 13 PNH patients on active complement inhibition who received mRNA SARS-CoV-2 vaccine from March 2021 at our center (23).

**Table 2:** Hemolytic flares in patients with paroxysmal nocturnal hemoglobinuria after SARS-CoV-2 vaccine.

PNH type	Therapy	N° of patients	Vaccine	Time to BTH	Therapy for BTH	Outcome	Ref.
1)Classic PNH 2)AA PNH 3)AA 4)Classic PNH 5)Classic PNH 6)Classic PNH	1)treatment naïve 2)Ravulizumab 3)Ravulizumab and Danicopan (oral complement factor D inhibitor) 4)Ravulizumab 5)Ravulizumab 6)Ravulizumab and Danicopan	6	1)Pfizer 2)Pfizer 3)Moderna 4)Moderna 5)Pfizer 6)Pfizer	1)5 days after second dose 2)same day of first dose 3)same day of second dose 4)same day of second dose 5)No BTH 6)No BTH	1)Ravulizumab, LMWH 2)Ravulizumab 3)Ravulizumab, RBC transfusion 4)Ravulizumab 5)Ravulizumab 6)Ravulizumab	1) microvascular bowel thrombosis 2)Resolved 3)Resolved 4)Resolved 5) resolved 6) resolved	*Gerber et al., Blood 2021* (21)
Classic PNH	treatment naïve	1	Moderna	One day after receiving the second dose	Eculizumab + methylprednisolone + RBC transfusion ➔ Switched to Ravulizumab in the outpatient setting	Resolved	*Portuguese et al., Blood advances 2021* (22)
Classic PNH	Ravulizumab	1	Moderna	One day after receiving the second dose	Ravulizumab, antibiotics, rhEPO, LMWH	Resolved	*Fattizzo B et al., Am J Hematol 2021* (23)

PNH, paroxysmal nocturnal hemoglobinuria; AA, aplastic anemia; BTH, breakthrough hemolysis; RBC, red blood cells; rhEPO, recombinant human erythropoietin.

These experiences indicate that hemolytic exacerbations may occur in PNH patients after SARS-Cov-2 vaccine so that monitoring of blood counts and hemolytic markers in the week after vaccine doses is advisable. Patient education to recognize signs/symptoms of PNH activity is pivotal, particularly in naïve patients who may be at higher risk of developing thrombotic complications. Although Gerber et al. observed BTH episodes in patients receiving COVID-19 vaccine more than 4 weeks after last ravulizumab dose, hemolytic flares may occur as soon as the day after the last complement inhibitor administration, so that it is difficult to suggest a specific timing, although it would be advisable to give vaccinations closer to anti-complement therapy (21). Importantly, transfusion requirement was rarer after COVID-19 vaccine than during infection, and no fatalities were reported in PNH after vaccination, confirming that vaccine expected benefits still outweigh the risks. Additionally, the risk of complement activation after Neisseria meningitis vaccination, and leading to thrombosis in patients with PNH has been reported (24).

### CAD Exacerbations During COVID-19 Infection

#### Case Description

A 68-year-old female diagnosed with CAD in 2017 previously treated with steroids and rituximab with response, was admitted to hospital in April 2020 due to fever and shortness of breath. She complained of dark urine and jaundice during the previous 5 days. Swab and CT scan revealed SARS-CoV-2 pneumonia and laboratory tests showed severe anemia (Hb 5.1 g/dL) with increased LDH 2.1 xULN. Direct antiglobulin test was confirmed positive for complement at high titer and consumption of C3 and C4 was noted (C3 <77 mg/dL, C4 undetectable). The patient was managed with oxygen support, hydroxychloroquine, antibiotics, steroids, and LMWH for COVID-19. For CAD reactivation, the patient received a total of 6 RBC transfusions during a period of 27 days. The clinical picture progressively improved and hemolytic anemia recovered, thereby allowing steroid tapering (from 50 mg day of prednisone reduced by 10 mg week until 25 mg day, then by 5 mg week until stop in about 8 weeks).

#### Literature Review

Available literature regarding CAD development or reactivation during COVID-19 infection is summarized in **Table 3** (25–42). From March 2020, we found 20 reports of patients experiencing cold agglutinin mediated hemolysis after COVID-19 infection (11 males, 9 females, aged from 17 to 77 years). Only 2 patients had a previous history of CAD (30, 42) whilst the others were firstly diagnosed after SARS-CoV-2 infection. All but 2 patients experienced mild to severe respiratory involvement, thereby requiring steroids, hydroxychloroquine, antibiotics, antiviral, tocilizumab, and intubation. Concerning CAD treatment, 12 patients received RBC transfusions, 8 high dose steroids, and 3 rituximab. Two patients received plasma exchange, one for CAD and one for COVID-19 itself. Four patients died despite medical interventions. One fatality occurred in a patient who experienced severe COVID-19 disease with deep venous thrombosis of upper extremities and cerebrovascular disease (33). Given the known risk of thrombosis in patients with CAD, the authors speculated that this may have contributed to thrombosis and to the unfavorable outcome. Accordingly, other investigators have suggested that the development of autoimmune hemolysis may represent a risk factor for worse outcome in COVID-19 patients. Specifically, Algassim et al. reported that 14.7% of intensive care unit (ICU) patients with COVID-19, and 9% of non-ICU patients, had AIHA (46). These patients had significantly more severe courses with longer hospital stay as compared with anemic patients without AIHA. Although anemia and increased levels of LDH and other hemolytic markers may be nonspecifically observed during severe infections, autoimmune hemolysis should be suspected in the context of an unexplained or persistent anemia in patients with COVID-19. From a pathogenic point of view, molecular mimicry among SARS-CoV-2 antigens and red cells seem the prominent mechanism, as also described for CAD developing after Mycoplasma infection (47). Additionally, the hyperinflammation triggered by the virus itself may result in cross activation of the immune system against self-antigens in an “*innocent bystander*” fashion. This includes the complement cascade that may be activated through both the classic and lectin pathway. Furthermore, the oxidative stress against erythrocyte and platelet membranes typical of the septic state, may favor the exposure of phosphatidyl serine (PS) and consequent complement deposition, thus causing blood cell consumption (47–49).

**Table 3:** Hemolytic flares in patients with cold agglutinin disease (CAD) and warm autoimmune hemolytic anemia (wAIHA) during COVID-19 infection.

Study type	Population of CAD	N°	Time to AIHA	Clinical presentation	Covid treatment	Covid outcome	CAD treatment	CAD outcome	Ref
Case report	Adult (54y, M)	1	–	Pneumonia	Hydroxychloroquine, tocilizumab, plasma exchange	Resolved	Steroids, plasma exchange	Resolved	Ramos-Ruperto et al., SN Compr Clin Med 2021 (25)
Case series	Adult (62y, F)	3	4 days	Severe pneumoniae	Not reported	Resolved	Steroid, rituximab	Remission	Lazarian et al., Br J Haematol 2020 (26)
	Adult (69y, F)		10 days	Moderate pneumoniae			steroids	Remission	
	Adult (61y, M)		11 days	Mild pneumoniae			transfusion	Still active hemolysis	
Case report	Adult (49y, F)	1	Not reported	Severe pneumonia	Not reported	Not reported	Not reported	Not reported	Ahmadnezhad et al., Hematol Transfus Cell Ther 2020 (27)
Case report	Adult (46y, F)	1	Not reported	Pneumonia	Not reported	Death	Not reported	Death	Zagorski Br J Haematol 2020 (28)
Case report	Adult (51y, F)	1	Concomitant	Pulmonary embolism	Heparin	Resolved	transfusion	Remission	Patil et al., Hematol Oncol Stem Cell Ther 2020 (29)
Case report	Adult (70y, M)	1	Concomitant	Not reported	steroids	Resolved	Transfusion, plasma exchange	Remission	Ahmed et al., BMJ Case Rep 2021 (30)
Case report	Adult (24y, F)	1	3 days	Pneumonia	Favipiravir, darunavir/ritonavir, azithromycin	Resolved	None	Remission, spontaneous	Moonla et al., Clin Case Rep 2020 (31)
Case report	Adult (77y, M)	1	9 days	Pneumonia	Intravenous steroids, antibiotics, hydroxychloroquine	Death	Transfusion	NR	Gupta et al., Eur J Case Rep Intern Med 2021 (32)
Case report	Adult (48y, M)	1	Concomitant	DVT, cerebrovascular disease	ND	Death	Transfusion	NR	Maslov et al., TH Open 2020 (33)
Clinical trial	Adult (33y, F)	1	Concomitant	Pneumonia	Steroids, tocilizumab	Resolved	Transfusion, steroids, rituximab 600 mg single infusion	Remission after rituximab	Jacobs et al., Transfusion 2021 (34)
Case report	Adult (62y, M)	1	16 days	Pneumonia	Intubation	Resolved	Transfusion	Remission	Capes et al., Ann Hematol 2020 (35)
Case report	Adult (69y, F)	1	20 days	Not reported	Levofloxacin, steroids	Resolved	Steroids, rituximab, Intravenous immunoglobulins	Remission	Aldaghlawi et al., Clin Case Rep 2021 (36)
Case series	Adult (43y, F)	2	6 days	Pneumonia	Oxygen, antibiotics	Resolved	Transfusion	Remission	Huscenot et al., Ann Hematol 2020 (37)
	Adult (63y, M)						Not reported	Remission	
Case report	Adult (45 y, M)	1	Concomitant	Pneumonia	Not reported	Not reported	Transfusion	Not reported	Raghuwanshi, Cureus 2020 (38)
Case report	Adult (61 y, M)	1	21 days	Pneumonia	Intubation, hydroxychloroquine, azithromycin, methylprednisolone	Resolved	None	Spontaneous recovery	Kaur et al., Cureus 2020 (39)
Case report	Adult (17y, M)	1	Concomitant	Mild pneumonia	None	Resolved	Steroids, transfusion	Remission	Wahlster Pediatr Blood Cancer 2020 (40)
Case report	Adult (48y, M)	1	6 days	Severe pneumonia	mechanical ventilation, vasopressors, sedation	Resolved	Transfusion	Remission	Hassanein et al., J Med Cases 2021 (41)
Cohort study	Adult (71y, F)	1/108 CAD patients	4 days	Severe pneumonia	oxygen, hydroxychloroquine, azithromycine, lopinavir/ritonavir	Resolved	Steroids, transfusions	Remission	Barcellini et al., Front Immunol 2021 (42)
**Study type**	**Population of wAIHA**	**N°**	**Time to AIHA**	**Presentation**	**Covid treatment**	**Covid outcome**	**AIHA treatment**	**AIHA outcome**	**Ref**
Case report	Adult (54y, M)IgG+	1	–	Pneumonia, diabetic ketoacidosis, acute kidney injury, hematuria, and anemia	–	Resolved	Steroids	Resolved	Huda et al., Cureus 2021 (43)
Case report	Adult (33y, F)IgG+C+	1	–	Asymoptomatic	–	.	Transfusions, Steroids	Resolved	Liput et al., Cureus 2021 (44)
Case series	Adult (72y,F) IgG+	2/3	concomitant	Bilateral pneumonia in both	Hydroxychloroquine, dexamethasone and tocilizumab	Resolved	Transfusion, steroids	Resolved	Ramos-Ruperto et al., SN Compr Clin Med 2021 (25)
	Adult (76y,F) IgG+								
Case series	Adult (61y,M) IgG+C+	4/6	13 days	Moderate pneumonia	Oxygen, Steroids, hydroxychloroquine, lopinavir and ritonavir.	Resolved	Steroids and transfusions, rituximab in 1	Not resolved at the time of publication	Lazarian et al., Br J Haematol 2020 (26)
	Adult (89y,F) IgG+		7 days	Mild pneumonia					
	Adult (61y,M) IgG+		9 days	Severe pneumonia					
	Adult (75y,M) IgG+		6 days	Moderate pneumonia					
Case report	Adult (24y,M) IgG+C AIHA	1	concomitant	Fever, myalgias and cough, pulmonary embolism, encephalitis	Steroids, anticoagulants, intubation, vasopressors, intravenous immunoglobulin	Superimposed Cryptococcus neoformans infection and death	Steroids, cyclophosphamide	Partial remission	Woldie et al., J Med Cases 2020 (45)
Retrospective study	Adult (59y,M) IgG+	2/139	–	Bilateral pneumonia and dysimmune encephalitis	intubation, steroids, hydroxychloroquine, tocilizumab, darunavir, and LMWH prophylaxis, IvIg	Resolved	Nothing	Remission	Barcellini et al., Front Immunol 2020 (42)
	Adult (78y,M) IgG+		3 weeks	moderate pneumonia	oxygen support, steroids, HCQ, azithromycine, full-dose LMWH		Steroids and IVIG		
Case report	Adult (33y, F) IgG+C+	1	concomitant	Bilateral pneumonia	Steroids, tocilizumab	Resolved	Trasnfusion, steroids, rituximab	Remisson	Jacobs et al., Transfusion 2021 (34)

Overall, the association between CAD development/reactivation and COVID-19 infection has been reported with more than half cases showing severe anemia requiring transfusions. Interestingly, the proportion of COVID-19-associated CAD is greater than what generally observed with CAD among all AIHAs, where CAD represent about 20% of cases (5). Most cases resolved with steroids and along with resolution of COVID-19 infection, but thrombotic episodes may occur and require prophylaxis (as also suggested for COVID-19 infection itself). Finally, it is still debated whether rituximab can be safely administered during septic state, and in most cases this treatment was deferred to allow recovery of active infection (50).

### CAD Exacerbations After SARS-CoV-2 Vaccination

#### Case Description

A 59-year-old male suffering from CAD since 2019, and previously treated with rituximab, had an Hb drop from 10.1 to 6.8 g/dL and LDH elevation (1.8 xULN) 5 days after the second dose of Moderna vaccine. The levels of complement fractions were also decreased, with C3 55 mg/dL (normal values >77 mg/dL) and undetectable C4. He had also experienced fever and pain at the injection site and complained of dark urine and jaundice starting the day after vaccination. He received steroids (1 mg/Kg day prednisone) and recombinant erythropoietin (40,000 UI/week of epoetin alpha subcutaneously) and Hb stabilized at about 8.2 g/dL. Two weeks later, rituximab was administered with progressive and complete recovery, allowing steroid tapering.

#### Literature Review

As shown in **Table 4**, only 4 cases of CAD or mixed form of AIHA (i.e., direct anti-globulin test positive for IgG plus C at high titer) developing (N=2) or reactivating (N=2) after SARS-CoV-2 vaccination have been reported (23, 28, 51, 52). Al Aoun et al. reported a 45-year-old female patient developing severe CAD 3 days after the first dose of Pfizer vaccine. The patient was treated with blood transfusion and rituximab, achieving complete remission 8 weeks later (51). Brito et al. reported an 88-year-old woman who developed very severe AIHA (Hb 4.5 g/dL) complicated by acute kidney injury two days after the second dose of an mRNA vaccine (not specified). The DAT revealed very high titers of anti-erythrocyte IgG autoantibodies and high anti-C3d titers. The patient was treated with methylprednisolone 1 g boluses and was transfused with subsequent Hb stabilization and amelioration of hemolytic markers (52). As regards exacerbations of pre-existing CAD, Lamas et al. reported a 57-year-old female who experienced shortness of breath, jaundice and mild hemoglobinuria 2 days after the first dose of an mRNA vaccine (not specified); hb had decreased to 8.6 g/dL and the patient received prednisone 20 mg daily with progressive improvement. The same episode occurred 2 days after the second dose of the same vaccine. Anti-Spike IgG testing showed that the patient had efficiently seroconverted (55). Interestingly, our group reported the results of a prospective monitoring of 58 individuals with AIHA, including 15 CAD during SARS-CoV-2 vaccination campaign. This consisted in testing Hb and LDH levels the week before and after each vaccine dose. We detected 3 warm AIHA and 1 CAD reactivation, all effectively managed with steroids and/or rituximab (23).

**Table 4:** Hemolytic flares in patients with cold agglutinin disease (CAD) and warm autoimmune hemolytic anemia (wAIHA) after SARS-CoV-2 vaccine.

Study type	Population	N°	Vaccine	Time to event	AIHA treatment	AIHA outcome	Ref
Case report	Adult (45y, F) CAD	1	Pfizer, 1^st^ dose	4 days	blood transfusions, rituximab	Remission	Al Aoun and Motabi (51)
Case report	Adult (88y, F) CAD	1	mRNA vaccine, 2^nd^ dose	2 days	Transfusions, methylprednisolone 1 g bolus	Remission	Brito et al. (52)
Case report	Adult (57y, F) CAD	1	mRNA vaccine, 1^st^ dose mRNA vaccine, 2^nd^ dose	2 days 5 days	prednisone 20 mg day	Remission	Zagorski et al. (28)
Cohort study	Adult (77y,M) CAD	1/15 CAD patients	Moderna vaccine, 1^st^ dose	7 days	Steroids, rituximab, recombinant erythropoietin	Remission	Fattizzo et al. (53)
Cohort study	Adult (79y,F) IgG+IgA+ wAIHA	3/41 wAIHA patients	Pfizer vaccine, 1^st^ dose	7 days	Steroids	Remission	Fattizzo et al. (53)
	Adult (73y,M) IgG+ wAIHA		Moderna vaccine, 1^st^ dose	7 days	Steroids		
	Adult (73y,M) IgG+wAIHA		Pfizer vaccine, 2^nd^ dose	5 days	Steroids		
Case report	Adult (41 y, F) IgG+ wAIHA	1	Moderna, 1^st^ dose	20 days	Transfusion, steroids, rituximab, mycophenolate mofetil, and immunoglobulins	Remission	Gadi et al. (54)
Case report	Adult (88y,F) IgG+C+ wAIHA	1	mRNA vaccine, 2^nd^ dose	2 days	Transfusions, steroids	Remission	Brito et al., Cureus 2021 (52)

The case described and data from literature show that SARS-CoV-2 vaccination may be associated with CAD development as well as with exacerbation in less than 10% of pre-existing CAD cases (i.e., 1/15 in our experience). CAD exacerbations are unpredictable, may occur after either after the first or the second dose, and regardless of vaccine type. Since most cases may be successfully managed with steroids and transfusion, prospective hematologic monitoring as adopted in our survey (1 week before, 1 week after the first and the second dose) appears appropriate to intercept and manage hemolytic flares. Similarly to what described for PNH, CAD episodes following COVID-19 vaccine are milder than those following infection, with no thrombotic nor fatal events, strengthening the message that the benefits of vaccination greatly outweigh the risks.

### Exacerbations/Development of wAIHA After COVID-19 Infection or SARS-CoV-2 Vaccination

Eleven patients with warm type AIHA developing or reactivating during COVID-19 infection have been reported in the literature (**Table 3**) (25, 26, 34, 42, 44, 45, 54). Of these patients, 4 showed complement positivity at DAT evaluation (26, 34, 44, 45). WAIHA flares occurred concomitantly with- or up to 21 days after initial COVID-19 detection and were mostly managed with transfusions and steroids. Only 2 patients required rituximab and 1 received cyclophosphamide. Notably, we previously reported 2 patients out of a cohort of 139 wAIHA patients who experienced a severe COVID-19 infection (42). Only one experienced wAIHA reactivation 3 weeks after COVID-19 pneumonia and was effectively managed with steroids and intravenous immunoglobulins. Additionally, 5 cases of wAIHA (only 1 had DAT positive for IgG and C3d) developing or reactivating after mRNA SARS-CoV-2 vaccination have been described, 3 after the 1^st^ and 2 after the 2^nd^ dose (**Table 4**) (23, 52, 54). Three cases had a severe presentation (i.e., Hb<8 g/dL), 2 required transfusions and only one subject received therapy other than steroids (rituximab and mycophenolate). Overall, as outlined for patients with CAD, hemolytic flares after COVID-19 infection appear more severe than those occurring after vaccination and the same monitoring of blood counts and LDH should be applied.

### Exacerbations/Development of HUS During COVID-19 Infection

The review of the literature showed a total of 9 patients with HUS associated with COVID-19 infection (**Table 5**) (56–59). Eight subjects developed an atypical HUS and 1 a typical HUS with bloody diarrhea in the preceding days and confirmed E. coli infection. Interestingly, most patients had mild or asymptomatic COVID-19 infection, whilst HUS presented with severe haemolytic anemia and acute kidney injury requiring transfusions, hemodialysis, and the C5 inhibitor eculizumab in 7 cases (56–59). All HUS flares occurred within 1 month from COVID-19 infection, mostly concomitantly, and all resolved. The largest cohort was that reported by El Sissy et al., who noted a sharp contrast between mild respiratory symptoms and severe renal and neurological HUS manifestations (54). Importantly, 3/5 subjects had undergone a previous renal transplant and were receiving tacrolimus/everolimus therapy that may have contributed to trigger HUS. Interestingly, patients with COVID-19- associated atypical HUS, who underwent genetic testing, were found to harbour genetic complement dysregulation, and 2 patients were positive for antibodies against complement factor H. Although limited, available data suggest that COVID-19 is a potential trigger for HUS, in accordance with previous data on complement-mediated aHUS precipitated by viral infections such as Influenza. The clinical suspicion should be acute to prompt quick diagnosis and establishment of supportive treatment (hemodialysis, transfusions and plasma exchange), along with use of anti-complement therapies.

**Table 5:** Hemolytic flares in patients with hemolytic uremic syndrome (HUS) during COVID-19 infection.

Study type	Population	N°	Time to AIHA	Clinical presentation	Covid treatment	Covid outcome	Hemolytic treatment	hemolytic outcome	Ref
Case series	5 patients with COVID-19-associated atypical HUS	5	Concomitant to 30 days	mild respiratory symptoms renal ysfunction, severe thrombocytopenia, neurological symptoms (confusion, central facial palsy), intestinal involvement (pain, diarrhoea), intestinal capillary thrombi.	Oxygen in 1/5 patients	Resolved	Two patients underwent plasma exchanges with fresh frozen plasma, while three were treated with eculizumab. Patient 4 received two infusions of rituximab for anti-FH antibodies.	All resolved	El Sissy et al., Blood 2021 (56)
Case series	Adult (22y,F) atypical HUS	2	Concomitant	Diarrhea, vomiting, loss of taste, fatigue, severe hemolytic anemia	–	–	Hemodialysis, Transfusions, plasma exchange, eculizumab	Both cases Resolved	Kaufeld et al., Kidney Int Rep 2021 (57)
	Adult (52y,F) atypical HUS		2 days	flu-like symptoms, loss of taset, fatigue, abdominal cramps, severe hemolysis			Ttransfusions, hemodialysis, eculizumab		
Case report	Adult (28y,F) atypical HUS	1	Concomitant	Fever, dysphagia, headache, hemolytic anemia, mild thrombocytopenia, acute kidney injury	–	–	Eculizumab, penicillin prophylaxis, anticoagulation	Resolved	Ville et al., Kidney Int 2021 (58)
Case report	Adult (24y,F) typical HUS	1	Concomitant	bloody diarrhea, acute kidney injury, and focal seizures	–	–	Eculizumab, lorazepam, levetiracetam, valproic acid	Resolved	Simpson et al., Epilepsy Behav Rep 2021 (59)

### Discussion and Conclusions

There is an association between COVID-19 and exacerbations of complement mediated hemolytic anemias. Although hemolytic flares in these diseases may be observed upon several infectious triggers (i.e., Mycoplasma pneumoniae, hepatitis, herpetic viruses, human immunodeficiency virus, Influenza virus, etc) (4, 60), and sporadically after other vaccines (i.e., Influenza vaccine) (61–63), SARS-CoV-2 seems to induce a higher and broader complement activation. Complement is clearly involved in the pathogenesis of COVID-19, namely respiratory failure, intravascular coagulopathy, and over-inflammation. Recently, it has been shown that a prominent activation of the alternative and lectin complement pathways identify a subset of severe COVID-19 patients with a higher proportion of fatalities, need of oxygen support, and ICU admission (64–67). If during COVID-19 infection alternative and lectin pathways are more involved, SARS-CoV-2 vaccine seems to mainly act through the classical pathway activation (21). As a matter of fact, complement activation has a dichotomous nature since it does contribute to the hyper-inflammatory state but may also exert a neutralizing effect against SARS-CoV-2 infection. This may account for the controversial results of ongoing trials with complement inhibitors (18, 19, 68–70). Likewise, immunosuppressive treatment in immune-mediated anemias may have a dual effect in reducing hyper-inflammation and dampening immunocompetence.

Patient education is pivotal to promptly recognize signs/symptoms of hemolytic flares and to seek medical attention. The latter should be always high and should focus on the assessment of the severity of hemolytic exacerbation to prompt treatment choice (transfusions, complement inhibitor initiation/additional dose in the case of PNH, steroids/rituximab in the case of CAD and wAIHA, plasma exchange, hemodialysis and complement inhibitor in the case of aHUS) and anti-thrombotic prophylaxis. Since most episodes occurred within the first 10 days after COVID-19 infection/vaccination, laboratory monitoring in that period appears feasible and cost-effective. Finally, hemolytic exacerbations occurring during COVID-19 infection were more severe, required higher therapeutic burden, and carried more complications including fatalities, as compared to those developing after SARS-CoV-2 vaccine. This highlights the importance of vaccinating this patient population but with meticulous monitoring for complications.

## Data Availability Statement

The raw data supporting the conclusions of this article will be made available by the authors, without undue reservation.

## Ethics Statement

The studies involving human participants were reviewed and approved by Comitato Etico Milano Area 2. The patients/participants provided their written informed consent to participate in this study.

## Author Contributions

All authors equally contributed to the present article.

## Conflict of Interest

The authors declare that the research was conducted in the absence of any commercial or financial relationships that could be construed as a potential conflict of interest.

## Publisher’s Note

All claims expressed in this article are solely those of the authors and do not necessarily represent those of their affiliated organizations, or those of the publisher, the editors and the reviewers. Any product that may be evaluated in this article, or claim that may be made by its manufacturer, is not guaranteed or endorsed by the publisher.
